# Cerebellar mitochondrial dysfunction coincides with structural and behavioral abnormalities in 3q29Del mice

**DOI:** 10.64898/2026.09.25.754481

**Published:** 2026-09-26

**Authors:** May K Kretzer, Elizabeth Arnulfo Candelario, Ryan Montalvo, Xinyan Leng, Jonathan A Coello, Zihui Ou, Aaron E Pozo-Aranda, Jennifer G Mulle, Zhen Yan, Ryan H Purcell, Meike E van der Heijden

## Abstract

3q29 deletion (3q29Del) is a genetic risk variant for autism spectrum disorder and schizophrenia that often results in developmental delays, cognitive disability, and impaired fine motor function. People with 3q29Del have reduced cerebellar volume, which correlates with symptom severity, and many 3q29Del-associated phenotypes also commonly occur after cerebellar injury or dysfunction. However, it is unknown whether the existing 3q29Del mouse model recapitulates the cerebellar dysfunction observed in humans. To characterize cerebellar phenotypes and uncover pathological differences in the 3q29Del mouse model, we investigated cerebellar structure, motor and vocal behaviors, protein expression, and mitochondrial function. We found uniformly reduced cerebellar volume in 3q29Del mice. Behavioral assays revealed vocal impairments in 3q29Del pups, fine motor impairments in adult mice, and reduced social mating calls in adult male mice. Proteomic analysis revealed enrichment of synaptic and mitochondrial proteins among the differentially expressed proteins in 3q29Del cerebellum tissue. Furthermore, mitochondria from 3q29Del mouse cerebellum displayed reduced oxygen flux and increased electron leak. These results recapitulate many human 3q29Del phenotypes in the 3q29Del mouse model and indicate mitochondrial dysfunction as a potential driver of 3q29Del pathology. Our findings also point to cerebellar involvement in 3q29Del phenotypes and provide a foundation for further research on cerebellar development in 3q29Del.

## Full Text Availability

The license terms selected by the author(s) for this preprint version do not permit archiving in PMC. The full text is available from the preprint server.

